# Evidence, trust, and objectivity with generative AI: a qualitative interview study of pre-service science teachers’ truth-assessment practices

**DOI:** 10.3389/fpsyg.2026.1781772

**Published:** 2026-04-29

**Authors:** Hongyu Hu, Siliang Yu, Lijun Xu

**Affiliations:** 1 School of Education, Mianyang Teachers' College, Mianyang, China; 2 Faculty of Education, Sichuan Normal University, Chengdu, China; 3 Faculty of Management, Shinawatra University, Bang Toei, Pathum Thani, Thailand

**Keywords:** epistemic cognition, generative AI, hallucination, pre-service science teachers, trust calibration, verification

## Abstract

Generative artificial intelligence (GenAI) systems can present information in a persuasive, scientific register while still producing subtle errors or making unsupported claims. For pre-service science teachers (PSTs), this creates a practical dilemma: determining what is sufficiently reliable to use, particularly when the intended audience is future students. This qualitative interview study examined how 20 PSTs enrolled in a science teacher education program at a public undergraduate university in Sichuan Province, China, evaluated the truthfulness of GenAI-style explanations. Participants completed a vignette-based think-aloud task and a semi-structured interview exploring evidence standards, trust calibration, conceptions of objectivity, and verification stop rules. Using Framework Analysis, we identified five themes: (1) situational evidence standards and locally embedded authority infrastructures; (2) trust calibration shaped by familiarity, perceived risk, and time pressure; (3) dual conceptions of objectivity—as rhetorical neutrality and as a justificatory process; (4) hierarchical verification strategies with explicit thresholds for “enough checking”; and (5) prudent instructional decisions under epistemic uncertainty, including revising, qualifying, or rejecting GenAI outputs. These findings position truth assessment as a situated professional practice rather than a decontextualized skill. They also suggest priorities for teacher education: making verification routines teachable, foregrounding objectivity-as-process, and connecting AI literacy to pedagogical responsibility.

## Introduction

Generative artificial intelligence (GenAI) systems, including large language models, have shifted from novelty to everyday infrastructure in education. Pre-service teachers (PSTs) now encounter GenAI not only as a writing assistant but also as a “knowledge partner” that can draft explanations, generate examples, and propose lesson ideas within seconds (Giannakos et al., 2025; Kasneci et al., 2023). At the same time, policy guidance has emphasized that GenAI use in education is inseparable from professional responsibility. Educators are expected to leverage efficiency and creativity without outsourcing judgments about what is reliable, fair, and pedagogically appropriate (UNESCO, 2023; U.S. Department of Education, Office of Educational Technology, 2023).

A central challenge is that GenAI can sound authoritative even when it is incorrect. Because many language models optimize for generating plausible text rather than ensuring factual grounding, they can “hallucinate”—producing confident statements, explanations, or citation-like references that are unsubstantiated or fabricated (Ji et al., 2023; Kasneci et al., 2023). In science teacher education, this matters because science teaching is not only about conveying accurate content; it also involves modeling how knowledge claims are warranted through evidence, argumentation, and transparent links between assertions and what can be checked (Osborne and Pimentel, 2023).

This dilemma also unfolds within a broader “post-truth” environment in which people routinely encounter competing science-related claims through social media, search engines, and persuasive media genres. Schooling must therefore prepare learners to evaluate claims, sources, and evidence under conditions of uncertainty and strategic communication (Chinn et al., 2021; Osborne and Pimentel, 2022). Research on public understanding of science similarly shows that many science-related judgments rely on trust in institutions and expertise, particularly when individuals cannot personally reproduce relevant evidence (Howell and Brossard, 2021). GenAI intensifies this tension by generating science-like discourse at scale while obscuring provenance.

Research on GenAI in teacher education suggests that PSTs are already incorporating these tools into learning and planning routines while also expressing concerns about inaccuracy, overreliance, and erosion of academic integrity (Karataş and Yüce, 2024; Yang and Appleget, 2024). Related concerns about the effectiveness and ethical use of AI tools in academic writing have also been documented among PhD scholars (Subaveerapandiyan et al., 2025). In science teacher education, emerging evidence indicates that PSTs may plan to integrate GenAI into inquiry-based teaching, increasing the urgency of understanding how they decide what is “safe to use” when GenAI outputs become potential instructional resources (Ramnarain et al., 2024). However, much of the early literature has emphasized adoption (e.g., intentions, perceived usefulness) rather than the practical epistemic work PSTs must perform when outputs are uncertain.

This gap is consequential for science education at the present moment. When GenAI outputs are fluent, plausible, and easily adaptable for classroom use, the central risk is not only that PSTs may encounter inaccurate information, but that they may normalize forms of knowledge use that are weakly warranted, poorly traceable, or insufficiently checked before being passed on to students. In science teaching, this matters especially because teachers do not simply transmit content; they also model what counts as a justifiable claim, what kinds of evidence are worth consulting, and when uncertainty should be disclosed rather than hidden. If teacher education focuses mainly on adoption, efficiency, or willingness to use GenAI, it may underprepare PSTs for this practical epistemic work and inadvertently treat verification as optional rather than integral to responsible teaching. For this reason, understanding how PSTs judge whether GenAI content is credible, defensible, and “safe to teach” is not a peripheral issue but an urgent question for science teacher education.

This study addresses that gap by examining PSTs’ truth-assessment practices with GenAI. We conceptualize truth assessment not as a single fact-checking step, but as a situated practice in which PSTs attend to credibility cues, invoke evidence standards, select verification actions, and decide when to stop checking under constraints and varying stakes. Using semi-structured interviews and a vignette-based think-aloud task, we studied 20 undergraduate PSTs at a public institution in Mianyang, Sichuan Province (pseudonym: University M). The design foregrounds the kinds of decisions PSTs anticipate making in practice: whether an output is trustworthy for personal learning and whether it is responsible to use (or adapt) for teaching.

Guided by this purpose, the study asks: (1) What counts as “evidence” for PSTs when they judge GenAI outputs, and how do thresholds for evidential sufficiency vary by task and stakes? (2) How do PSTs calibrate trust in GenAI across topic familiarity, perceived risk, and time pressure? (3) How do PSTs construe objectivity in GenAI outputs—particularly the distinction between objective-sounding style and objectivity as a process of transparency and checkability? (4) What verification repertoires and stopping rules do PSTs use, and how do these shape decisions about whether content is “safe to teach”?

By examining the everyday logic of truth assessment, this study contributes to science teacher education in three ways. Empirically, it documents how PSTs reason through GenAI-related uncertainty in classroom-relevant contexts. Conceptually, it integrates evidence, trust, and objectivity within a single analytic lens for understanding AI-mediated epistemic judgment. Practically, it identifies teachable leverage points, particularly around source traceability, trust calibration, and the ethics of using AI-generated knowledge in instruction.

## Literature review

### GenAI in education and the problem of plausible unreliability

Recent scholarship highlights GenAI’s potential to support learning and teaching by rapidly generating explanations, examples, questions, and feedback (Giannakos et al., 2025; Kasneci et al., 2023). Yet the same fluency and speed that make GenAI attractive also introduce epistemic risk. Because language-model outputs are shaped by statistical regularities rather than disciplined commitments to truth, evidence, and traceability, models may produce misinformation or unjustified claims (Bender et al., 2021; Ji et al., 2023). In educational contexts, this risk can be amplified by pragmatic pressures (e.g., completing tasks quickly), which may tacitly relax verification standards.

Policy documents therefore convey a dual message: GenAI can be productive, but educational use requires strengthened norms for transparency, verification, and accountability (UNESCO, 2023; U.S. Department of Education, Office of Educational Technology, 2023). These norms are especially salient for classroom-facing applications, where teachers remain responsible for instructional quality and harm prevention.

### Epistemic cognition: how people decide what counts as “good enough to know”

To understand truth assessment with GenAI, it is useful to draw on epistemic cognition—the study of how people evaluate knowledge claims, including what they treat as evidence and what standards they apply for justification (Chinn et al., 2011). Epistemic cognition is particularly relevant in contexts where individuals must judge claims under uncertainty, incomplete information, and competing authorities (Chinn et al., 2021). A key implication is goal sensitivity: standards for “good enough” justification often shift with purpose, such as learning efficiently versus teaching responsibly.

In science education, epistemic judgment is shaped by disciplinary norms. Learners are expected to value explanations that are constrained by evidence, attentive to boundary conditions, and open to revision. Recent scholarship argues that contemporary information environments require learners to act as “competent outsiders”—individuals who cannot reproduce specialized scientific work but can still evaluate claims by attending to credibility, evidence quality, and the integrity of knowledge-producing institutions (Osborne and Pimentel, 2023). GenAI complicates this task by simulating disciplinary discourse while obscuring provenance.

### Trust calibration and appropriate reliance on AI systems

Truth assessment with GenAI involves more than detecting errors; it also requires calibrating trust—deciding when reliance is warranted and when skepticism is necessary. Classic work on trust in automation argues that trust is functional when it is aligned with system capabilities and limitations, rather than uniformly high or uniformly low (Hoff and Bashir, 2015; Lee and See, 2004). Human–computer interaction research similarly shows that trust calibration is shaped by interface cues and user beliefs, particularly when systems produce uncertain outputs (Wischnewski et al., 2023). These dynamics matter for GenAI because confident language, stepwise reasoning, or citation-like detail can function as reliability cues even when content is not grounded (Ji et al., 2023).

In teaching, trust calibration also has an ethical dimension. For teachers, the costs of error extend beyond personal inconvenience to student misunderstanding and potential erosion of professional credibility. Trust decisions, therefore, operate as judgments about professional responsibility under uncertainty.

### Objectivity as style versus objectivity as a checkable process

A recurring risk in GenAI use is equating objectivity with a particular linguistic style (e.g., neutral tone, balanced phrasing). Science education scholarship, however, treats objectivity less as a rhetorical posture and more as a social–epistemic practice grounded in transparency, contestability, and the capacity to check claims against evidence and sources (Osborne and Pimentel, 2023). Because GenAI can produce objective-sounding prose while remaining epistemically opaque, PSTs’ objectivity judgments provide a useful window into what cues they treat as warrant.

The concept of epistemic vigilance is informative here. People routinely monitor communication for reliability using cues about competence and plausibility (Sperber et al., 2010). In GenAI contexts, however, familiar cues are destabilized: there is no human speaker with an inspectable track record, and competence can be linguistically simulated. Examining PSTs’ objectivity judgments can therefore reveal which cues they import from human communication and which new heuristics they develop for AI-mediated claims.

### Verification repertoires and “stopping rules” under time and stakes

Truth assessment is enacted through concrete verification actions—what people check, where they look, and how they decide to stop. Research on online information evaluation shows that effective verification often involves lateral reading: leaving the initial text to investigate sources and corroboration across independent sites (Breakstone et al., 2021; McGrew, 2024; Wineburg and McGrew, 2019). Educational research further suggests that these strategies are teachable and can improve how learners manage uncertainty (McGrew and Breakstone, 2023). For PSTs, verification may also rely on discipline-specific resources (e.g., textbooks, curriculum standards, reputable science organizations) and pedagogical considerations (e.g., whether an explanation could plausibly mislead students).

Crucially, verification is shaped by stopping rules—practical judgments about what counts as “enough checking.” PSTs may adopt stricter stopping rules for teaching than for private learning because classroom use activates duty of care and reputational risk. Accordingly, this study treats verification as part of a broader practice that links evidence standards, trust calibration, and objectivity judgments.

## Methods

### Research design

We used an interpretive qualitative design to examine how pre-service science teachers (PSTs) assess the truthfulness of generative AI (GenAI) outputs that may appear plausible yet be partially ungrounded or fabricated. Data were generated through semi-structured interviews combined with a vignette-based think-aloud task. This pairing enabled us to elicit participants’ everyday, locally situated rules for evaluating GenAI outputs and to observe those rules across comparable scenarios (Kvale and Brinkmann, 2009; Patton, 2015). We conceptualized truth assessment as a situated professional practice in which PSTs attend to cues, apply evidence standards, calibrate trust, select verification actions, and enact stopping rules under constraints and varying stakes.

### Setting and participants

Participants were 20 PSTs enrolled in an undergraduate science teacher education program at a public institution in Sichuan Province, China (pseudonym: University M). We used purposive sampling to recruit PSTs who were actively engaged in teacher preparation and who could describe—or plausibly anticipate—GenAI use in science learning and lesson preparation. Participants were assigned IDs (P01–P20). To reduce deductive disclosure in a single-institution sample, we report participant characteristics in broad categories rather than fine-grained personal details. Table 1 summarizes the participant context and reporting conventions (Year 1: 18–19; Year 2: 19–20; Year 3: 20–21; Year 4: 21–22).

**Table 1:** Participant characteristics (University M; *n* = 20).

**Characteristic**	**Reporting approach**
Institutional context	Undergraduate science teacher education program at University M (Sichuan, China; pseudonym)
Participant group	Pre-service science teachers (PSTs), undergraduate
Year level and age bands	Year 1 (18–19), Year 2 (19–20), Year 3 (20–21), Year 4 (21–22)
Identifiers	P01–P20; identifying details removed or generalized
Confidentiality strategy	Broad categories and de-identification to minimize deductive disclosure in a single-site study

The sample size was determined purposively in relation to the study’s focused analytic aim rather than statistical representativeness. Because the study examined a relatively specific phenomenon—PSTs’ truth-assessment practices with GenAI—using repeated interviews structured around common focal constructs and a shared vignette task, 20 participants provided sufficient depth for cross-case and cross-vignette comparison. During analysis, later interviews primarily added variation, nuance, and boundary cases to the existing thematic framework rather than introducing wholly new analytic domains, which supported the adequacy of the sample for this interpretive purpose. We therefore treat sample sufficiency in terms of analytic and meaning saturation within a focused qualitative design, while recognizing that this does not imply exhaustiveness across all PST populations. Participants were all enrolled in an undergraduate science teacher education program; however, to reduce deductive disclosure in this single-site study, the de-identified dataset retained only broad participant categories rather than fine-grained disciplinary specialization. We therefore do not make discipline-specific claims and now acknowledge this as a limitation of the study.

### Data collection

#### Interview protocol

We followed a semi-structured protocol organized around the study’s focal constructs: evidence criteria, trust calibration, objectivity-as-practice, verification repertoires, and thresholds and trade-offs. The protocol (Appendix A) began with warm-up questions about prior GenAI experiences and typical use cases. It then proceeded through four sections: (1) mapping everyday use scenarios; (2) a critical incident narrative involving suspected inaccuracy or misleading output; (3) explicit elicitation of evidence criteria and source hierarchies; and (4) prompts about trust calibration and objectivity, including whether objective-sounding language was treated as a credibility cue. Semi-structured interviewing ensured common coverage while preserving space for participants’ practical reasoning and trade-off management (Kvale and Brinkmann, 2009; Patton, 2015).

#### Vignette think-aloud task

To observe truth assessment under comparable conditions, participants completed a vignette task in which they reviewed three short GenAI-style responses and were invited to think aloud about what they trusted, what they doubted, what they would verify first, and what would count as “enough verification” before using content—particularly for teaching. Think-aloud tasks are well suited for eliciting reasoning processes while enabling structured probing (Ericsson and Simon, 1993). The vignettes varied in the form of uncertainty: V1 contained a subtle conceptual issue; V2 used confident language but provided weak support and omitted key boundary conditions; and V3 included citation-like detail, including at least one reference designed to be difficult to verify quickly. Vignette texts and probes are provided in Appendix A.

#### Vignette provenance and design rationale

The vignette texts were researcher-designed stimuli rather than verbatim outputs from a single GenAI system. They were written in a GenAI-like register to simulate recurrent epistemic features that participants may plausibly encounter in educational use: a fluent explanation containing a subtle conceptual weakness (V1), a confident pedagogical recommendation with weak warrant and missing boundary conditions (V2), and citation-like detail that invites traceability checking but may not be readily verifiable (V3). This design choice was intentional. The aim of the vignette task was not to evaluate the behavior of a particular model, interface, or prompting condition, but to hold the epistemic challenge relatively constant across participants so that their truth-assessment reasoning could be compared under common conditions. The texts were reviewed and lightly refined for clarity, length, and disciplinary plausibility prior to use. Accordingly, the ecological relevance of the vignettes lies in their simulation of common GenAI-like epistemic risks rather than in representing the exact output profile of any single system.

#### Procedure and data management

Interviews lasted approximately 50–75 min and were audio-recorded with participant consent. The vignette task was administered during the interview using screen share or printed handouts. Recordings were transcribed verbatim and de-identified. The research team also wrote brief post-interview notes to capture contextual cues relevant to interpretation. Data were stored on password-protected devices, with access restricted to the research team.

Interviews were conducted and transcribed in Mandarin Chinese. For reporting, excerpts were translated into English with attention to conceptual equivalence rather than literal word-for-word correspondence (Squires, 2009; Temple and Young, 2004; van Nes et al., 2010). A bilingual researcher reviewed translated excerpts against the Mandarin originals, and discrepancies were resolved through discussion with reference to the original recordings. Because the data were collected in Mandarin, we also treated key evaluative terms as context-sensitive analytic constructs rather than assuming one-to-one lexical equivalence in English. In participants’ talk, expressions corresponding to “objectivity” did not always appear as a single stable term; some participants instead used language closer to “accuracy,” “scientificness,” or “sounding scientific.” In analysis, these expressions were interpreted in context and grouped according to the function they served in participants’ reasoning—for example, whether they referred to stylistic neutrality, source traceability, checkability, or a more general sense of correctness. The English labels used in this article therefore represent analytic categories grounded in the Mandarin data, rather than direct word-for-word translations of a single vernacular term.

### Data analysis

#### Analytic approach

We analyzed data using Framework Analysis, which is well suited to applied qualitative studies requiring transparent procedures and systematic cross-case comparison (Gale et al., 2013; Ritchie and Spencer, 1994). This approach supported consistent attention to evidence, trust, and objectivity across interviews while enabling matrix-based comparisons across participants and across vignette tasks.

#### Framework development and indexing

We began with an initial deductive framework aligned with the research questions and operationalized through the code families in Appendix B (e.g., credibility cues/red flags; evidence criteria; trust calibration; conceptions of objectivity; verification repertoire; thresholds/trade-offs; teaching actions). The framework served as an organizing structure for indexing and charting, while allowing inductive refinement at the level of sub-codes and analytic memos when participants introduced locally meaningful distinctions. Transcripts were coded in meaning units, and segments could receive multiple codes. Domain-level categories were kept stable to preserve comparability across cases (Gale et al., 2013; Ritchie and Spencer, 1994).

#### Theme development and safeguards against circularity

To reduce the risk that research-question–guided indexing would predetermine the findings, we treated the initial framework as an open organizing structure rather than a closed codebook. We added inductive sub-codes when participants introduced distinctions not anticipated in the initial matrix and refined code definitions through constant comparison across cases. We also conducted deviant-case analysis to qualify theme boundaries and to specify conditions under which decision steps were abbreviated, reordered, or omitted. Themes therefore reflect a synthesis of research-question–guided organization and inductively derived differentiation within themes.

#### Charting, comparison, and interpretation

After indexing, we charted data into two matrices (Appendix B): (a) a cross-case thematic matrix (participant × domain) and (b) a vignette comparison matrix (participant × vignette × key constructs). Each cell contained a concise analytic summary and a link back to the corresponding transcript segment to preserve traceability. We then examined patterns across participants and tasks, focusing on: (1) which cues triggered verification, (2) what counted as “evidence” and “enough evidence,” (3) how trust thresholds shifted with familiarity, time pressure, and teaching stakes, and (4) how objectivity was construed as style versus process, and how these construals shaped subsequent action.

Coding was led by a primary researcher using the framework described above, with ongoing audit and peer-debriefing support from the research team. The balance between deductive and inductive work was as follows: domain-level categories remained aligned with the research questions to preserve cross-case comparability, while sub-codes, distinctions, and memoed interpretations were refined inductively when participants introduced unanticipated but analytically meaningful patterns. To strengthen trustworthiness, selected transcripts, charted summaries, and theme boundaries were reviewed within the research team. Where interpretations differed, discrepancies were discussed with reference to the original Mandarin transcript, the corresponding charted matrix entry, and the evolving code definitions until a defensible analytic formulation was reached.

### Trustworthiness and ethics

Credibility was strengthened through within-interview triangulation: everyday-use mapping, critical incidents, and vignette reasoning offered complementary windows into participants’ practices (Patton, 2015). We maintained an audit trail (framework versions, coding memos, and matrix iterations) to support dependability and confirmability (Lincoln and Guba, 1985; Nowell et al., 2017). During analysis, we attended to disconfirming cases, so themes were grounded in variation rather than only in modal accounts. Ethical procedures followed institutional requirements. Participants provided informed consent, were reminded that participation was voluntary and that they could withdraw at any time, and were assured that reporting would use pseudonyms and de-identification. All participants were adult university students, and written informed consent was obtained directly from each participant.

## Findings

In the interviews and the vignette think-aloud task, PSTs described truth assessment with GenAI as more than “checking facts.” What they narrated was a situated practice: noticing cues, deciding what counts as evidence, calibrating trust, selecting verification actions, and deciding when to stop—often with an additional layer of responsibility when the intended use was teaching. Five themes capture these patterns and illustrate how “evidence,” “trust,” and “objectivity” were enacted as local rules in science teacher preparation. Where relevant, we also note minority patterns that help delineate the boundaries of these local rules.

### Theme 1 (RQ1): evidence standards shift with stakes—"good enough for me” versus “safe to teach”

PSTs did not treat “evidence” as a fixed requirement. In low-stakes personal learning, many relied on conformity with prior learning plus internal coherence: if a response aligned with textbook ideas or class notes and “made sense,” they were willing to use it with minimal scrutiny. P03 (Year 3; 20–21) summarized this logic: “For self-study or review, if AI’s explanation matches the textbook’s core points and reads smoothly, I’ll use it… no need for excessive scrutiny.” Here, evidence was largely treated as compatibility with prior learning plus a coherent line of reasoning. However, a minority described applying a stricter baseline even for self-study, treating coherence as insufficient and routinely cross-checking against textbooks or trusted websites before adopting an explanation.

That standard changed sharply when PSTs spoke about teaching preparation. For intended classroom use, participants emphasized that evidence must be traceable and defensible because teaching makes the teacher publicly accountable for claims. P14 (Year 4; 21–22) framed multi-source checking as non-negotiable: “Teaching is not personal learning… AI-generated teaching content needs… two or three authoritative checks,” such as textbooks, curriculum standards, or core academic sources. In these accounts, evidence functioned as professional anchoring to curricular authorities and reputable sources, sufficient to withstand challenge. Even within teaching-facing talk, a few PSTs framed “defensibility” as a pragmatic minimum (e.g., alignment with curriculum standards plus one authoritative source) rather than exhaustive confirmation, citing feasibility constraints in school settings.

Between these ends, medium-stakes work (e.g., assignments) often elicited an intermediate standard in which PSTs scaled evidence investment to perceived consequence. P08 (Year 2; 19–20) described revising content against the textbook for routine work but verifying AI-cited claims in academic databases for graded papers or teaching demonstrations. Overall, Theme 1 indicates that PSTs’ truth assessment was not a single epistemic stance toward GenAI, but a differentiated set of evidence thresholds shaped by use context.

### Theme 2 (RQ2): trust is calibrated through familiarity, perceived responsibility, and time constraints

Participants rarely described trust as a stable trait (“I trust AI”/“I do not trust AI”). Instead, trust was something they adjusted in response to what they knew, what was at stake, and what time they had. Topic familiarity shaped baseline trust: for familiar foundational topics, PSTs felt they could quickly spot problems and therefore used GenAI as a supportive explanation tool with selective checking. P12 (Year 3; 20–21) explained: “For topics like photosynthesis, I spot errors instantly… I trust it more and only verify key details.” For unfamiliar or emerging topics, participants reported a sharp drop in baseline trust and a corresponding increase in verification effort; some described treating GenAI as an initial draft requiring substantial external validation before it could be relied upon (e.g., P06, Year 2; 19–20). Some participants also described beginning from external sources irrespective of familiarity, using GenAI mainly to rephrase or organize ideas once facts were established.

Perceived responsibility—especially teaching responsibility—raised trust requirements beyond what was acceptable for personal use. Many PSTs framed this as an ethical distinction between personal consequences and student consequences, including actively searching for counterexamples and boundary conditions even when an answer seemed plausible. P02 (Year 4; 21–22) captured this logic: “Mistakes in personal use only affect me, but teaching misinformation harms a class… even perfect AI answers need a second check… Are there exceptions?” Time pressure shaped verification pathways without eliminating minimum commitments for teaching: participants described simplifying steps while preserving core checks. As P17 (Year 3; 20–21) noted, under last-minute constraints they would cross-verify core points with textbooks or school resources, and if unconfirmed they would label content “for reference only” rather than teach it as fact. In a few accounts, when verification could not be completed within time or access constraints, PSTs managed risk by avoiding GenAI use for teaching rather than relying on partially checked content.

### Theme 3 (RQ3): objectivity is construed in two ways—objectivity-as-style versus objectivity-as-process, with some participants reporting shifts after “being fooled”

When PSTs talked about “objectivity,” two contrasting conceptions appeared, and these conceptions shaped how participants interpreted GenAI’s scientific tone. Some initially treated objectivity as presentation: neutral language, structured formatting, and technical vocabulary were taken as signals that an answer was “scientific” and therefore credible. P15 (Year 1; 18–19) described trusting “objective-sounding” answers until they found that “its cited research was fake.” In this view, objectivity could be inferred from surface cues, and verification could remain relatively superficial until an inconsistency was noticed. Conversely, a few participants treated stylistic “objectivity” as a warning sign, describing overly polished neutrality as a cue to verify more rather than less.

In contrast, other PSTs defined objectivity as a practice of checkability: transparency about sources, clear reasoning, and the ability to withstand scrutiny. They rejected the idea that polished style guarantees truth and instead prioritized traceability and accessible original evidence. P07 (Year 4; 21–22) reflected that formal AI writing can “use jargon without explaining principles,” and described shifting toward “traceability: clear sources, rigorous logic, and accessible original evidence.” Several participants linked this process-oriented stance to prior episodes in which outputs sounded authoritative but proved unreliable. P09 (Year 3; 20–21) summarized the shift: “Now I judge ‘correctness,’ not ‘presentation,’ and demand verifiable evidence.” Taken as retrospective accounts rather than observed developmental change, these narratives suggest that objectivity is negotiated as a professional norm when PSTs make sense of uncertainty in GenAI-like outputs. Some did not readily invoke “objectivity” as a category at all, speaking instead in terms of “accuracy” or “scientificness,” suggesting that the construct was not equally salient across participants even when similar verification practices were described.

In interpretive terms, the distinction reported here is therefore not a claim that participants uniformly possessed or used a single vernacular equivalent of “objectivity.” Rather, the analytic contrast between objectivity-as-style and objectivity-as-process was developed from how participants used related evaluative language in context: some treated scientific-sounding neutrality, formal tone, or careful wording as signs of credibility, whereas others emphasized that claims should be traceable, checkable, and open to scrutiny. We retain the term “objectivity” as an analytic label because it captures the conceptual contrast at stake, while recognizing that participants themselves sometimes framed the issue through adjacent terms such as “accuracy” or “scientificness.” One extended excerpt illustrates how some participants moved from treating objectivity as a matter of style to treating it as a matter of process:

*When I first started using GenAI for lesson preparation, I mainly judged it by how it wrote. If it used professional scientific terms, had a neutral tone, and explained things step by step, I took that as a scientific and objective answer. I even used some of that content directly in micro-teaching at first, until my supervisor pointed out that one explanation about cell respiration had no clear theoretical basis, even though it sounded very professional. That experience made me realize that sounding objective and scientific is far from enough. GenAI can use jargon to package vague reasoning, and its writing style can make a weak or even wrong point sound convincing. Now, when I judge whether an output is really objective and usable, I focus first on traceability: does it point to sources I can actually check, such as textbook chapters, authoritative scientific papers, or curriculum standards? Second, checkability: can I verify the reasoning with other reliable resources, and is there concrete evidence for the claims? Third, scrutinizability: if a student asks why something is true, can I take them back to the original evidence and let them examine it for themselves? If an output cannot meet these three conditions, then no matter how objective it sounds, it is only empty language for teaching.* (P07, Year 4).

This excerpt makes visible the inferential shift that was less apparent in shorter quotations: stylistic neutrality was not dismissed entirely, but it was re-ranked as insufficient unless supported by traceable sources, checkable reasoning, and claims that could withstand classroom scrutiny.

### Theme 4 (RQ4): verification repertoires form a tiered escalation—screening, triangulating, and scholarly confirmation when red flags appear

PSTs reported a repertoire of verification strategies and a tendency to escalate verification as uncertainty or stakes increased. At a basic level, they began with quick screening: checking whether an explanation conflicts with prior knowledge, whether key terms align with textbooks/notes, and whether the reasoning “hangs together.” P11 (Year 2; 19–20) described this as a fast check of “formulas and steps,” with a textbook cross-check for key terms—aimed at removing obvious errors rather than proving absolute truth. Not all participants described escalation beyond this stage: some reported stopping after screening even for nontrivial tasks due to limited time or access, and then managing residual uncertainty by reusing only general ideas while withholding precise details, strong claims, or citation-like elements.

For more consequential or less familiar tasks, PSTs described moving toward triangulation across sources, often combining reputable science websites, comparison with other tools, and consultation with peers. P04 (Year 3; 20–21) described verifying an uncertain claim via a reputable popular-science site, cross-checking with another educational AI, and consulting groupmates before using the content. When the stakes were highest—particularly teaching demonstrations, public-facing lessons, or unfamiliar interdisciplinary topics—participants described scholarly verification and human consultation, including checking citations via DOIs and attending to study limitations before incorporating claims into a plan (e.g., P18, Year 4; 21–22). Some also emphasized social verification (e.g., asking mentors or experienced teachers) as their highest tier, indicating that escalation sometimes ended in human judgment rather than documentary confirmation.

Escalation was frequently triggered by “red flags,” such as missing boundary conditions, absolute language, or citation-like details that felt unverifiable. P13 (Year 2; 19–20) described treating words like “always” or incomplete citations as alarms that prompt immediate checking for exceptions or source authenticity. Across cases, these triggers help explain why the same PST might accept a GenAI explanation for self-study yet reject or heavily verify similar outputs for classroom use. In a few instances, participants described pre-emptive escalation even without explicit red flags when the topic implicated safety or contentious claims, treating stakes alone as sufficient to trigger higher-tier checking.

### Theme 5 (RQ4, teaching-facing): teaching decisions are prudent—modify, disclose, or refuse, with an added goal of modeling critical thinking

When PSTs moved from truth assessment to teaching decisions, they drew a strong boundary between what feels “safe for myself” and what is “safe for students.” Many described GenAI as an auxiliary tool rather than a content authority. Even when the core idea seemed acceptable, direct classroom use was rare; participants preferred to modify content, add boundary conditions, and make provenance visible. However, participants differed in whether they would explicitly disclose GenAI involvement: some preferred to minimize mention of GenAI in class, whereas others treated transparency as part of responsible modeling. A second extended excerpt shows how participants distinguished between content that was acceptable for personal understanding and content that was safe to teach:

*For this GenAI explanation of why the sky is blue, I think it is acceptable for my own review and self-study. Its basic idea about light scattering is correct, and it is easier to understand than the textbook, so for personal use I could take the general point without checking every detail. But that is never enough for teaching students, and I would not use it directly in class. First, it mentions nitrogen in the atmosphere but leaves out oxygen’s role in scattering, which creates a subtle conceptual gap. Even if that seems minor, students could come away with an incomplete understanding. Second, as a teacher, I am responsible for every word I use in class, so using unpolished content would risk misleading students. Before teaching with it, I would need to do at least two further steps: cross-check it against the high school science textbook and the provincial science curriculum standards, and then verify the scattering principle through a reputable science website, such as one run by the Chinese Academy of Sciences. After that, I would revise the explanation, mark in my lesson plan that this section drew on GenAI but was checked and corrected, and, if appropriate, even show students how the original AI answer contained a small but important omission. If I do not have time to verify and revise it in this way, I would rather not use it at all.* (P17, Year 3).

Here, the participant’s reasoning moves beyond truth evaluation alone. Verification, revision, source disclosure, and the possibility of withholding the material altogether were treated as parts of a single responsibility-oriented judgment about whether the content was safe to teach. When content was partially useful but not fully verifiable, some PSTs described disclosure-based use: making uncertainty explicit and turning verification into a learning opportunity. Responding to the vignette that included citation-like detail, P05 (Year 4; 21–22) described using the general viewpoint while telling students that the specific research could not be verified “temporarily” and inviting the class to discuss how verification could be done.

Finally, when PSTs judged an output to be fundamentally wrong, weakly supported, or clearly misleading, refusal was described as the appropriate professional response. Reacting to the vignette suggesting repetitive practice is superior to inquiry, P01 (Year 1; 18–19) rejected the claim as contrary to constructivist teaching and “not supported by reliable research,” concluding they would not use it and would instead design the lesson themselves. Beyond immediate accuracy, several participants also described a student-development rationale: teaching decisions should model how to think with (and against) GenAI, not simply whether to use it. P16 (Year 3; 20–21) described deliberately raising questions—even when content seemed correct—so students learn to ask “Why did AI say this? Are there other explanations?” and to treat verification as part of scientific reasoning. A small number additionally described avoiding GenAI in assessed or public lessons, viewing reputational and ethical risks as too high even when verification seemed possible.

### A synthesis: PSTs’ “local rules” for truth assessment form a heuristic pathway

**Figure 1:**
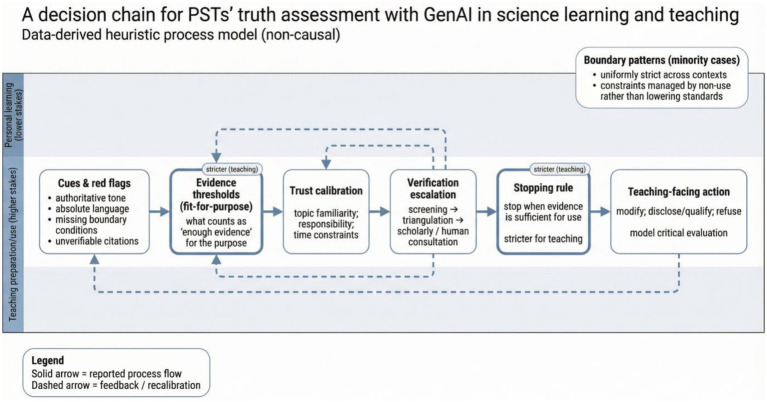
A heuristic, recursive pathway of PSTs’ reported truth-assessment practices with GenAI in science learning and teaching, integrating Themes 1–5. Solid arrows indicate commonly reported directional movement in participants’ reasoning, while dashed arrows indicate possible feedback, looping, or recalibration. The figure should be read as a heuristic synthesis of reported practices rather than as a fixed or causal sequence: participants sometimes abbreviated, skipped, reordered, or revisited steps depending on familiarity, stakes, time pressure, and intended classroom use. Teaching responsibility is shown as tightening evidence thresholds and stopping rules, and the boundary patterns summarize minority but analytically important variations.

Taken together, the themes suggest that PSTs’ truth assessment practices can be understood as a heuristic pathway: cues and red flags shape initial doubt or confidence; evidence standards are set relative to purpose and stakes; trust is calibrated by familiarity, responsibility, and time constraints; verification actions are selected and escalated as needed; and a stopping rule is applied that becomes noticeably stricter for teaching than for personal learning. Teaching functions as a responsibility multiplier within this chain, tightening what counts as “enough evidence” and encouraging prudent teaching-facing actions (modification, disclosure, or refusal), alongside a growing interest in using GenAI as a context for modeling critical evaluation. Importantly, these local rules were not unanimous; the boundary patterns noted above show that some PSTs adopted uniformly strict standards across contexts, while others managed constraints by restricting use rather than escalating verification. We also conducted an exploratory cross-case review by year level, given that participants spanned Years 1–4 of the teacher-education program. This review did not suggest a simple linear progression from lower- to higher-year participants. Instead, year-related differences appeared partial and uneven. In several cases, participants in later years described somewhat more elaborated verification routines or more explicit concern with what would be professionally defensible in classroom use, which may reflect greater proximity to teaching practice. However, these tendencies were not uniform, and caution was evident in some lower-year participants as well. Overall, the data are better interpreted as showing patterned variation rather than a developmental sequence: verification depth, objectivity judgments, and stopping rules appeared to be shaped not only by year level, but also by topic familiarity, perceived stakes, and participants’ own responsibility framing. Figure 1 visualizes this synthesis as a data-derived heuristic (non-causal) process model, making explicit how participants linked evaluative cues, evidence thresholds, trust calibration, verification escalation, stopping rules, and teaching-facing actions across contexts.

To clarify what counted as a more adequate truth-assessment move in the vignette task, we also examined, at a descriptive level, whether participants’ reported reasoning actually engaged with the epistemic challenge embedded in each case. In V1, adequacy involved noticing that an apparently fluent scientific explanation still contained a conceptually incomplete or potentially misleading point for teaching, rather than accepting coherence alone as sufficient. In V2, adequacy involved challenging the absolutist recommendation, noticing the missing boundary conditions and weak warrant, and resisting direct classroom uptake without further support. In V3, adequacy involved treating citation-like detail as a cue for verification rather than as self-authenticating evidence, for example by checking whether references were searchable, traceable, and pedagogically defensible. Read in this way, the heuristic pathway did not only describe what PSTs said they would do; it also showed where their judgment was more or less responsive to different kinds of embedded epistemic risk. Because the study was designed to elicit reported reasoning rather than to administer a formal performance test, these patterns should be interpreted as descriptive indicators of practical epistemic adequacy within the interview task rather than as direct measures of generalized competence.

## Discussion

This study examined how pre-service science teachers (PSTs) judge “what is true” when generative AI (GenAI) can sound convincing yet be incorrect. Across themes, truth assessment emerged as a situated practice in which PSTs set evidence thresholds, calibrate trust, interpret “objectivity,” and select verification actions, with the most pronounced shifts occurring when participants anticipated classroom use. Building on this integration, Figure 1 synthesizes the findings into an interpretive, data-derived heuristic pathway of reported reasoning. It should not be read as a fixed sequence of steps or a causal model. Rather, it captures a recurrent pattern in how participants described moving among cues, evidence thresholds, trust calibration, verification actions, and teaching-facing decisions, while also allowing for abbreviated, skipped, reordered, and recursive paths. In the Discussion below, we use this heuristic pathway to (a) clarify how “fit-for-purpose” evidence standards operate under different stakes, (b) connect trust calibration and verification escalation to science-education accounts of epistemic practices and warranted reliance on authority, and (c) articulate teaching-facing implications (e.g., modify, disclose/qualify, refuse, and modeling critical evaluation).

### Situational differences in evidence standards: contextual selection of authority sources (RQ1)

Theme 1 shows that PSTs’ evidence standards functioned as conditional thresholds rather than fixed rules. When GenAI outputs “felt like science,” participants often began with authority heuristics—who produced the claim and whether the apparent source aligned with local expectations for scientific legitimacy. This was not simple deference; instead, it resembled a pragmatic “competent outsider” stance that acknowledges inevitable reliance on epistemic authorities while seeking to make that reliance context-sensitive and defensible (Osborne and Allchin, 2025; Osborne and Pimentel, 2023). In our sample, authority selection was typically anchored in familiar infrastructures (e.g., textbooks, curriculum standards, institutionally endorsed websites, teacher educators), which served as practical bridges from uncertainty to action.

These patterns should also be interpreted in light of the study’s local teacher-education setting. The participants were enrolled in a public undergraduate teacher-education program in Sichuan Province, where judgments about what is “safe to teach” are likely shaped by a recognizable authority ecology: the centrality of textbooks and curriculum standards, the practical importance of institutionally endorsed resources, and the expectation that future teachers be able to justify instructional choices in ways that are professionally defensible. In such a context, verification is not only about factual correctness in the abstract; it is also about alignment with pedagogical norms, anticipated classroom accountability, and the risk of misleading students under conditions where authoritative curricular materials remain highly salient. This may help explain why participants often treated textbooks, official standards, and teacher educators as especially important anchors when calibrating trust in GenAI outputs. At the same time, because these findings come from a single-site study, this institutional pattern should be read as a situated interpretive condition rather than as a universal feature of PSTs’ authority judgments across contexts.

GenAI complicates evidential judgment because it can mimic surface markers of authority—confident tone, technical vocabulary, and citation-like formatting—without performing the epistemic work those markers usually imply (Bender et al., 2021; Ji et al., 2023). Under these conditions, the goal of teacher education is not to eliminate trust, but to strengthen discrimination between warranted and unwarranted claims, especially when evidence is incomplete or ambiguous (Barzilai and Chinn, 2020; Chinn et al., 2021). Theme 1 adds a pedagogical nuance: PSTs did not only ask whether a claim sounded credible, but whether it was credible enough for a specific teaching context and audience.

### Three-dimensional influencing factors of trust calibration: familiarity, risk, and time pressure (RQ2)

Theme 2 frames trust as a calibrated, situational judgment rather than a stable attitude toward GenAI, consistent with accounts of appropriate reliance as task- and context-sensitive (Hoff and Bashir, 2015; Lee and See, 2004). Participants’ accounts clustered around three recurring conditions—topic familiarity, perceived risk and professional responsibility (especially when teaching was anticipated), and time pressure—describing how they adjusted reliance and verification under each condition. These patterns should be interpreted as situated practical reasoning rather than as causal “influences” with quantifiable effect sizes.

This matters because debates about GenAI in education often collapse trust into two endpoints—overreliance versus rejection—whereas policy guidance increasingly emphasizes use-with-guardrails, transparency, and verification norms (Miao and Holmes, 2023; U.S. Department of Education, Office of Educational Technology, 2023). PSTs’ narratives make this middle position more concrete. GenAI was often treated as a convenient starting point, yet participants anticipated circumstances in which convenience and limited feedback could relax standards and shift stopping rules, echoing concerns about “trust drift” in everyday use (Giannakos et al., 2025; Kasneci et al., 2023). A teacher-education implication is to make trust calibration teachable as routines: recognizing contexts that invite relaxed standards and adopting practicable stopping rules that preserve defensibility when stakes rise.

### Dual construction of objectivity conceptions: style-based neutrality and process-based objectivity (RQ3)

Theme 3 clarifies why GenAI can be persuasive in science education settings. PSTs distinguished between objectivity-as-style (neutral tone, careful qualifiers, impersonal voice) and objectivity-as-process (justification that can be traced, checked, and challenged). GenAI is often adept at producing the style of objectivity while remaining vulnerable to factual error and missing boundary conditions (Bender et al., 2021; OpenAI, 2023). Science communication and science education scholarship similarly cautions that objectivity is not guaranteed by rhetorical form; it is constructed through norms of evidence, critique, and argument evaluation (Howell and Brossard, 2021; Valladares, 2022). Our findings show PSTs negotiating this distinction in a teacher-education context where authoritative style can be mistaken for epistemic warrant.

This distinction is directly relevant to misinformation concerns, because the primary threat is often a plausible claim presented in scientific-looking language without epistemic accountability (Osborne and Pimentel, 2023). For teacher education, Theme 3 therefore suggests a concrete learning target: supporting PSTs to move from “objectivity as appearance” to “objectivity as disciplined method,” aligned with calls to teach how scientific knowledge is warranted, contested, and stabilized (Erduran, 2022; Osborne et al., 2022). GenAI makes this shift urgent by amplifying stylistic fluency while obscuring provenance and the conditions under which claims hold.

### Hierarchical characteristics of verification strategies: stop rules and “enough checking” (RQ4)

Theme 4 captures a pragmatic verification repertoire. PSTs did not verify everything; they verified strategically and escalated checks when uncertainty or stakes increased. This aligns with what is known about hallucination: fluent but unfaithful content in which errors may be subtle rather than conspicuous (Ji et al., 2023; Maynez et al., 2020). In educational settings, “verify all claims” is not actionable. The teachable question is what counts as enough verification for the task at hand. Our findings indicate tiered strategies that moved from quick screening (e.g., cross-searching key terms) to stronger checks (e.g., consulting authoritative texts, triangulating across reputable sources, and scrutinizing references when citation-like details appeared questionable).

This tiered pattern aligns with policy guidance that emphasizes verification and transparency while recognizing classroom time constraints (Miao and Holmes, 2023; U.S. Department of Education, Office of Educational Technology, 2023). It also resonates with calibrated reliance research: when feedback about system accuracy is limited, users may over-trust confident outputs unless they have explicit calibration supports (Hoff and Bashir, 2015; Wischnewski et al., 2023). A teacher-education implication is to frame verification as a pedagogical routine—akin to checking misconceptions or anticipating student questions—so it is professionally meaningful rather than positioned as an added moral burden.

### Prudence in teaching decisions under epistemic uncertainty: reframing GenAI as a pedagogical object (RQ4)

Theme 5 extends RQ4 by focusing on teaching-facing stopping rules and “safe to teach” judgments under plausible uncertainty. In science teacher education, the decision is not only “Is this true?” but “Can I responsibly teach with it?”—a distinction that becomes acute when information guides action (Chinn et al., 2021; Osborne and Pimentel, 2023). PSTs’ prudence—rewriting explanations, adding provenance notes or boundary conditions, disclosing uncertainty, or declining use—can be read as emerging professional norms of epistemic responsibility oriented toward protecting learners and maintaining instructional integrity.

A further implication is that GenAI can be treated not only as a production tool but also as a pedagogical object that makes epistemic judgment visible and learnable. Contemporary science literacy increasingly includes media and information competencies for evaluating claims, sources, and uncertainty (Howell and Brossard, 2021; Osborne et al., 2022). GenAI can concretize this agenda by foregrounding the tension between plausible explanation and reliable warrant, creating opportunities to model verification and uncertainty talk in classroom-relevant ways (Osborne and Allchin, 2025; Valladares, 2022).

### Contributions, limitations, and next steps

Taken together, the themes shift attention from whether teachers “trust” GenAI to how trust becomes professionalized through situated evidence practices. They also provide teacher educators with a compact instructional vocabulary—evidence thresholds, objectivity-as-process, and “enough verification”—that can be taught and assessed as routines. This orientation complements work highlighting reliance risks under convenience and weak feedback and calling for explicit calibration supports (Giannakos et al., 2025; Kasneci et al., 2023). It also aligns with AI literacy syntheses that foreground evaluation, verification, and responsible use as core components of AI-related competence in education (Zhou et al., 2025).

Several limitations bound the scope of these claims. First, the interview- and vignette-based design in a single institutional context captures PSTs’ reported reasoning and may overstate carefulness relative to real-time lesson-planning pressures. In addition, because participant characteristics were retained in broad categories for confidentiality, the de-identified dataset did not support fine-grained analysis by subject specialization (e.g., physics, chemistry, biology), so discipline-specific variation remains an important question for future research. Future work could pair interviews with artifact-based analysis, structured performance tasks, or logged interactions to observe verification moves directly. Second, because the vignettes used GenAI-style responses to elicit reasoning, this study does not evaluate the accuracy, citation validity, or other performance characteristics of any specific GenAI system. Third, as GenAI tools and interfaces evolve rapidly, replication across cohorts and platforms is needed to distinguish stable professional norms from tool-contingent responses (Miao and Holmes, 2023; OpenAI, 2023). Extending sampling to in-service teachers would further clarify how school accountability, curriculum pacing, and classroom experience reshape evidence thresholds and stopping rules.

Despite these caveats, the findings are timely. They show PSTs navigating truth assessment as both an epistemic and an ethical practice. This duality—knowing what to trust and deciding what is responsible to teach—may be among the most consequential professional learning outcomes for science teachers in the age of generative AI.

## Conclusion

This study examined how pre-service science teachers (PSTs) assess the truthfulness of GenAI-like outputs—fluent, persuasive explanations that may nonetheless contain subtle errors or weak warrants. In interviews and vignette-based think-alouds, PSTs did not treat truth assessment as a single act of “fact-checking.” Instead, they described a heuristic and sometimes non-linear heuristic pathway: noticing credibility cues and red flags, selecting task-appropriate evidence standards, calibrating trust based on familiarity and perceived risk, escalating verification when uncertainty increased, and applying stopping rules that became markedly stricter when the intended use was teaching.

Two implications follow. First, the most consequential boundary was not whether PSTs used GenAI, but whether they could justify claims derived from GenAI-like outputs through traceable sources and defensible reasoning. When classroom responsibility was salient, many participants shifted from relying on objective-sounding language to treating objectivity as a checkable process grounded in corroboration, boundary conditions, and transparency. Second, PSTs’ practices identify concrete leverage points for science teacher education: teaching tiered verification routines, supporting calibrated reliance rather than blanket trust or rejection, and positioning GenAI-like outputs as teachable artifacts for modeling how scientific knowledge is warranted.

Overall, responsible use of GenAI in science education may depend less on policing tools and more on strengthening professional judgment. This includes teachable routines for when to verify, when to stop, and when to reject—often through recursive or abbreviated decision paths—so that future teachers can decide not only what is plausible, but what is safe, evidence-based, and ethically teachable.

## Data Availability

The raw data supporting the conclusions of this article will be made available by the authors, without undue reservation.
