# Supplementary material for: Evidence, trust, and objectivity with generative AI: a qualitative interview study of pre-service science teachers’ truth-assessment practices
Source: Front Psychol. 2026 Apr 29;17:1781772. doi: 10.3389/fpsyg.2026.1781772 (PMC13170594; doi:10.3389/fpsyg.2026.1781772)
Supplement: Supplementary file 1 [file Supplementary_file_1.docx]

**Appendix A. Semi-Structured Interview Protocol and Vignette Task**

**A1. Opening script (2 minutes)**

Thank you for joining. This interview is about how pre-service science teachers judge whether GenAI outputs are trustworthy and “true enough” to use for learning or for teaching preparation. This is not a test—we are interested in your reasoning. Participation is voluntary. You can skip any question or stop at any time. With your permission, we will audio-record and transcribe the interview. Your identity will be protected through pseudonyms and de-identification.

**A2. Warm-up: background and GenAI experience (5 minutes)**

1. What year are you in (Year 1–4)?
2. In what ways have you used GenAI tools (if at all)? For example: learning science content, drafting explanations, lesson planning, assessment preparation, writing.
3. How often do you use GenAI in a typical week?
4. What do you usually use GenAI for in science-related tasks?

**A3. Use mapping: typical tasks and stakes (8–10 minutes)**

1. Think about the last few times you used GenAI for a science-related purpose. What were you trying to do?
2. When you use GenAI, is it usually for your own learning, for assignments, or for preparing to teach?
3. How do your expectations change when the output might be used for teaching (e.g., a lesson explanation for students)?
4. When time is limited, what changes in how you evaluate the output?

**A4. Critical incident: when you suspected an output might be wrong (10–12 minutes)**

1. Can you recall a time when a GenAI response seemed inaccurate, misleading, or incomplete?
2. What triggered your doubt (e.g., a detail that felt “off,” missing conditions, overly confident tone, strange citation)?
3. What did you do next, step by step?
4. What did you learn from that experience, and did it change your trust in GenAI?

**A5. Evidence criteria and source hierarchy (10–12 minutes)**

1. When you decide whether a claim is true, what counts as evidence for you?
2. What kinds of sources do you trust most in science (e.g., textbook, lecture notes, reputable organizations, peer-reviewed papers, teachers/mentors)? Why?
3. If a GenAI response includes citations, how do you interpret that? When do citations increase your trust, and when do they not?
4. What does “enough evidence” look like for: your own learning, versus; teaching or preparing materials for students?

**A6. Trust calibration (8–10 minutes)**

1. What is your default trust level in GenAI outputs (high/cautious/low)? Why?
2. What factors most increase your trust (topic familiarity, source links, logical coherence, style, past success)?
3. What factors most decrease your trust?
4. How does your trust change when the topic is unfamiliar?
5. How does your trust change when the stakes involve students?

**A7. Objectivity prompts (6–8 minutes)**

1. When people say a science explanation is “objective,” what do they usually mean?
2. Do GenAI responses sometimes sound objective? What makes them sound that way?
3. Can something sound objective but still be wrong? Can you share an example (AI or non-AI)?
4. What does “being objective” require in practice when you are preparing to teach science?

**A8. Vignette task: truth assessment “in action” (20–25 minutes)**“Next I’ll show you three short GenAI-style responses, similar to what you might encounter when learning or preparing to teach. Please think aloud. Tell me what you trust, what you doubt, and what you would do next. Again, this isn’t a test—we’re interested in your reasoning.”

**Vignette 1 (V1): Plausible explanation with a subtle conceptual issue**

GenAI-style response (show to participant):

“*The sky looks blue mainly because sunlight interacts with tiny molecules in Earth’s atmosphere. Shorter wavelengths of light scatter more easily, and blue light is scattered in many directions, so we see blue from all parts of the sky. At sunrise and sunset, sunlight travels through more atmosphere, so most of the blue light is scattered out of our line of sight and the remaining light appears red or orange. Some people think oxygen “adds” blue color to the sky, but the key reason is how light scatters in the air.*”
(Note: In this response, one statement is subtly misleading or incomplete in a way that matters for teaching. Participants are not told which statement.)

**Probes**

1. What’s your first impression—would you accept this as correct? Why?
2. Which parts feel trustworthy, and which parts make you pause?
3. What would you verify first, and how would you verify it?
4. Threshold probe: What would count as “enough verification” before you would teach this?

**Vignette 2 (V2): Confident tone but weak evidence / missing boundary conditions**

**GenAI-style response (show to participant):**

“*If you want students to remember a science concept, the best method is always to give them a clear, step-by-step explanation and then have them repeat it until it becomes automatic. This works for all topics because repetition strengthens memory. So, for example, if students struggle with chemical reactions, repeating balanced equations will reliably improve understanding. This is a proven approach and is more effective than inquiry activities, which often confuse learners.*”

**Probes**

1. What makes this response feel convincing (if anything)?
2. What is missing for it to count as “well supported” in your view?
3. If you had limited time (e.g., lesson tomorrow), what would be a “safe enough” way to handle this output?
4. Trade-off probe: What are you trading off here—speed, accuracy, clarity, or responsibility to students?

**Vignette 3 (V3): Citation-like detail with at least one unverifiable reference**

GenAI-style response (show to participant):

“*Research shows that using short “prediction questions” before an experiment increases conceptual change because it activates students’ prior knowledge and creates cognitive conflict (Li & Morgan, 2021). A meta-analysis of 42 studies reported moderate gains in science achievement when prediction prompts were used (Nguyen, Patel, & Cho, 2019). In addition, classroom trials found that prediction questions improved students’ explanations of evidence and reduced misconceptions (Zhang, 2020). Therefore, teachers should include prediction prompts in most lab lessons to improve learning outcomes.
References:
Li, Y., & Morgan, T. (2021). Prediction prompts and conceptual change in secondary science. Journal of Classroom Inquiry, 18(2), 55–72.
Nguyen, R., Patel, S., & Cho, J. (2019). Prediction questions in science labs: A meta-analysis. International Review of Science Learning, 11(1), 1–28.
Zhang, H. (2020). Prediction questions and evidence-based explanations in middle school labs. Teaching Science Quarterly, 9(3), 101–118.*”

**Probes**

1. Do the citations increase your trust? Why or why not?
2. How would you verify the citations? What counts as successful verification for you?
3. If you cannot find a reference, how would you interpret that? What would you do next?
4. Teaching-use probe: Would you still use any part of this content? If yes, which part and how would you label or frame it for students?

**Cross-vignette synthesis (4–6 minutes)**

1. Across these three cases, what patterns do you notice in your own judgment?
2. What are your biggest “red flags” that consistently trigger verification?
3. Are there situations where you would use GenAI even if you’re not fully sure? What makes that acceptable?

**Closing reflections: implications for teacher education (5–8 minutes)**

1. What should teacher education programs do to better prepare PSTs to judge GenAI outputs (truth, evidence, trust, objectivity)?
2. If you had to give one practical rule to a junior PST about using GenAI responsibly in science learning/teaching, what would it be and why?
3. Is there anything we did not ask that you think matters for understanding how PSTs assess truth with GenAI?

**Appendix B. Coding Manual (Framework Analysis)**

**B1. Purpose and analytic logic**

This coding manual operationalizes a Framework Analysis for the study Evidence, Trust, and Objectivity with Generative AI: A Qualitative Interview Study of Pre-Service Science Teachers’ Truth-Assessment Practices. The coding system supports (a) consistent indexing across participants and (b) matrix-based charting for systematic cross-case and cross-vignette comparison (Ritchie & Spencer, 1994; Gale et al., 2013). The framework provides an organizing structure aligned to the study’s research questions, while allowing inductive refinements as sub-codes or analytic memos when participants introduce locally meaningful distinctions.

**B2. Unit of coding and indexing rules**

**Unit of coding (meaning unit):** the smallest segment expressing a complete analytic idea (typically 1–5 sentences).
**Multiple coding:** apply multiple codes when a segment contains multiple analytic functions (cue + criterion + action + teaching decision).
**Include:** reasons for belief/doubt; definitions of evidence/trust/objectivity; verification actions; stopping rules; trade-offs; differences between “safe for me” vs “safe to teach.”
**Exclude:** filler talk; tool descriptions without judgment relevance.

**B3. Mandatory co-coding rules (consistency safeguards)**

Any mention of classroom/student use → C2c AND at least one P code.

Any “enough checking/minimum/stop checking/safe” language → TH1 and/or TH2 (add TH3–TH5 if trade-offs/responsibility appear).

If “objective/neutral/scientific-sounding” is used to justify trust → O1 (and O5 if conflation with truth is implied) + Q2.

Distinguish cue vs criterion vs action: Q = cue; E = evidence criterion; V = verification action.

**B4. Code families and definitions (compact codebook)**

**C. Context of use**

C1 Task type (learning, lesson planning, explanation, labs, assessment, writing)

C2a Low-stakes (personal learning/brainstorm)

C2b Medium-stakes (assignment/submission)

C2c High-stakes teaching (students/classroom)

C3 Time pressure/constraint

C4a/C4b Topic familiarity/unfamiliarity

**K. Hallucination awareness**

K1 Recognizes fabrication risk

K2 Assumes inherent accuracy (misconception)

**Q. Credibility cues & red flags**

Q1a/Q1b Confidence boosts trust / triggers caution

Q2 Scientific style cue (jargon, textbook tone, formal structure)

Q3 Logical coherence cue

Q4 Prior-knowledge alignment cue

Q5 Missing boundary conditions red flag

Q6a/Q6b Citations boost trust / citation skepticism

Q7 Suspicious specificity red flag

**E. Evidence criteria (RQ1 core)**

E1 Traceability to original source

E2 Authority-based evidence

E3 Cross-source triangulation

E4 Empirical/observable evidence

E5 Explanatory adequacy (mechanism-based)

E6 Curriculum/textbook alignment for “safe to teach”

E7 Citation verification standard (indexed/searchable/DOI)

E8 Evidence sufficiency threshold (purpose-dependent)

**H. Source ecology**

H1 Source ranking/hierarchy

H2 Database/search infrastructure (Scholar/library portals)

**T. Trust calibration (RQ2 core)**

T1a/T1b/T1c High/cautious/low baseline trust

T2 Conditional trust by task

T3 Conditional trust by familiarity

T4 Trust updating from experience

T5 Teaching raises trust requirements

T6 Overtrust pattern

T7 Undertrust/avoidance

**O. Objectivity conceptions (RQ3 core)**

O1 Objectivity-as-style (neutral tone = objective)

O2 Objectivity-as-process (checkability/transparency/critique)

O3 Objectivity-as-consensus/authority

O4 Objectivity critique (neutral ≠ true)

O5 Style–truth confusion

**V. Verification repertoire (RQ4 core actions)**

V1 Quick plausibility check

V2 Check textbook/lecture notes

V3 Reputable web check

V4 Scholarly verification (Scholar/databases/DOI)

V5 Human verification (mentor/peer/instructor)

V6 Ask GenAI for sources/limitations

V7 Counter-prompt testing (self-critique/counterargument)

V8 Rewrite with uncertainty / add boundary conditions

V9 Reject / do not use

**TH. Thresholds & trade-offs (RQ4 mechanisms)**

TH1 Minimum verification threshold

TH2 Stopping rule (“good enough”)

TH3 Time–accuracy trade-off

TH4 Usefulness–certainty trade-off

TH5 Responsibility framing (duty to students)

**P. Teaching actions**

P1 Use directly

P2 Use with modification

P3 Meta-use for teaching (critique/fact-checking activity)

P4 Disclosure to students

P5 Refuse classroom use

**S. Social/program influences**

S1 Program norms/instructor expectations

S2 Mentor influence

S3 Peer influence

**AF. Affective responses**

AF1 Anxiety/fear of being wrong

AF2 Confidence/empowerment

AF3 Frustration/distrust after errors

**B5. Charting matrices (required outputs)**

B5.1 Cross-case thematic matrix (Participant × Domain)
Rows: P01–P20; Columns: C | Q | E/H | T | O | V | TH | P | (optional S/AF).
Each cell includes: (1) 2–5 line charted summary; (2) 1–2 key phrases; (3) anchor to transcript; (4) data-source tag (critical incident vs vignette).

B5.2 Vignette comparison matrix (Participant × Vignette × Key Constructs)
For each participant and vignette (V1–V3), chart: Q cues/red flags; E/H criteria; T shift; V actions; TH threshold/stopping; P teaching decision.

**B6. Workflow (minimum reporting standard)**

Familiarization + case memos

Indexing with codebook + analytic memos

Charting into both matrices

Mapping/interpretation with attention to pattern variation and disconfirming cases

Audit trail: framework versions, memo log, and matrix iterations (Lincoln & Guba, 1985; Nowell et al., 2017)
